# Examination of clinical factors affecting intrauterine microbiota

**DOI:** 10.1530/RAF-20-0030

**Published:** 2021-02-02

**Authors:** Kei Odawara, Ryosuke Akino, Akihiko Sekizawa, Miwa Sakamoto, Seo Yuriko, Kanako Tanaka, Mutsumi Mikashima, Masami Suzuki, Yasushi Odawara

**Affiliations:** 1 Obstetrics and Gynecology in the Department of Surgery, Showa University, Shinagawa-ku, Tokyo, Japan; 2 Obstetrics and Gynecology, Showa University, Shinagawa-ku, Tokyo, Japan; 3 Fertility Clinic Tokyo, Shibuya-ku, Tokyo, Japan

**Keywords:** chronic endometritis, implantation failure, intrauterine microbiota, *Lactobacillus*, menstrual cycle

## Abstract

**Purpose:**

Following reports of an increase in implantation and pregnancy continuation rates by a higher percentage of *Lactobacillus* in the intrauterine microbiota, it has received attention in infertility treatment. This study aimed to examine Japanese women for intrauterine microbiota.

**Methods:**

The clinical background factors in women that influence the abundance of *Lactobacillus* in the bacterial microbiota were examined. We included 147 patients (31 and 116 in the follicular and luteal phase, respectively), from June 2018 to June 2020, who underwent their first intrauterine microbiota test and had not used antibiotics for at least 4 weeks before the test. In the luteal phase, we compared the background factors of women in cases with 90% or more and less than 90% of *Lactobacillus*. Differences in the intrauterine microbiota were examined during the follicular and luteal phases.

**Results:**

The proportion of *Lactobacillus* tended to be low among women aged 36 years and older with a history of childbirth (*P* = 0.0631). Some bacteria were only detected during the follicular and luteal phases, and the bacterial microbiota may change during the menstrual cycle.

**Conclusion:**

Bacterial microbiota in the uterus may differ between the follicular and luteal phases. Furthermore, it was shown that the rate of *Lactobacillus* may be lower in women (older than 36 years) who had given birth, indicating that intrauterine microbiological testing may be considered for these women in clinical practice.

**Lay summary:**

Good implantation and pregnancy continuation rates have been reported when the proportion of the bacteria *Lactobacillus* is high in the uterus (intrauterine) bacterial population (microbiota). In this study, we assessed whether the clinical background of Japanese women (age, history of pregnancy and childbirth, and presence of gynecological or hormonal disorders) affect the proportion of intrauterine microbiota. Intrauterine samples were collected and sequenced to evaluate the intrauterine microbiota and the composition ratio of each bacterium. Comparing the percentage of *Lactobacillus* in the latter phase of the menstrual cycle with the clinical background, it was found that the percentage tended to be lower in women with a history of childbirth. We compared the intrauterine microbiota between the first phase and latter phase of the menstrual cycle and revealed that it may differ between the two phases. Advances in the development of criteria for assessing intrauterine microbiota are expected.

## Introduction

The world’s first* in vitro* fertilization (IVF) baby was born in 1978, and the first IVF baby in Japan was born in 1983. The development of intracytoplasmic sperm injection (ICSI) has improved the pregnancy rate in assisted reproductive medicine, including tubal infertility patients and male infertility patients. Even if a healthy embryo is obtained by egg collection, there are many cases wherein implantation does not occur, and the pregnancy reaction becomes positive after transplantation; however, the result is a biochemical miscarriage. The causes may include the intrauterine environment, genetic abnormalities in the transferred embryo, oviductal edema, abnormal coagulation factors, and endocrine abnormalities, such as thyroid dysfunction. Conversely, it was previously thought that the uterus was sterile; however, advances in culture technology have reported the possibility of the presence of bacteria in the uterus.

Furthermore, in 2007, advances in next-generation sequencing (NGS) made it possible to quickly analyze the intrauterine microbiota by DNA analysis. In addition, the intrauterine microbiota comprising the intrauterine environment has been attracting attention (Moreno & Franasiak 2017, Baker *et al.* 2018). Moreno *et al.* reported that the bacterial microbiota in the vaginal and intrauterine areas was independent (Moreno *et al.* 2016, Moreno & Simon 2018). Furthermore, Chen *et al.* examined the bacterial microbiota present in the vaginal and fallopian tubes, cervix, intrauterine area, and intra-abdominal cavity. They reported that the microbiota at each site showed independent distribution (Chen *et al.* 2017). Moreno *et al.* reported that patients with intrauterine *Lactobacillus* percentages of more than 90% demonstrated significantly higher implantation and continued pregnancy rates than those with intrauterine *Lactobacillus* percentages of less than 90% (Moreno *et al.* 2016), and Kyono *et al.* confirmed this fact (Kyono *et al.* 2019). In response to this report, in recent years, an increasing number of institutions in Japan have proposed a test for uterine bacterial microbiota for cases in which good embryo transfer is performed once or more and implantation do not occur. However, there are no criteria for proposing intrauterine microbiota testing for patients of any clinical background. In this study, we examined clinical background factors, such as age and experience of embryo transfer, in Japanese women to clarify clinically recommended subjects for the examination of uterine microbiota. To determine the timing of the intrauterine microbiota examination within the menstrual cycle, changes in the intrauterine microbiota during the follicular and luteal phases were examined.

## Materials and methods

Overall, 147 Japanese patients aged 26–45 years who had their intrauterine microbiota examined for implantation failure at the Fertility Clinic Tokyo between June 2018 and June 2020 were included in the study. Patients with a history of examination and who had used antibiotics within 4 weeks were excluded from examination. The study was approved by the Fertility Clinic Tokyo’s ethics committee, and the included patients who were briefed on the study provided their consent. The follicular and luteal phases were determined from the last menstrual period and the change in the size of the principal follicle on ultrasound. After disinfection, an endosuction (open-ended type 2.5 × 3.0 × 250 mm, Hakko Co., Nagano, Japan) was carefully inserted into the uterus to avoid contact with the vaginal wall, and endometrial tissue was collected. The collected endometrium was immediately submitted to Varinos Corporation. The analysis of the intrauterine microbiota is based on the method described in previous report (Kyono *et al.* 2018). Briefly, the genimic DNA was extracted from the tissues. The variable region 4 (V4) hypervariable region of the bacterial 16S rRNA gene was amplified from the specimen’s DNA. The amplification product was sequenced by the Illumina MiSeq platform to determine the bacterial genome. The bacterial taxonomy were identified by referring to the Greengenes database v. 13_8.17, and the content of each genera in the intrauterine microbiota was analyzed. The group with more than 90% *Lactobacillus* in the uterus was defined as *Lactobacillus*-dominated microbiota (LDM), whereas the group with less than 90% was defined as non-*Lactobacillus*-dominated microbiota (NLDM). Factors that affect the uterine microbiota include age, embryo transfer history, pregnancy history, labor history, abortion history, chronic endometritis, endometriosis, endometrial polyp, the value of serum Anti-Müllerian hormone (AMH), and the presence or absence of high serum thyroid-stimulating hormone (TSH) levels (>2.50) were compared between the LDM and NLDM groups. When considering pregnancy rates, the same factors were similarly examined in the 26–35 and 35–45 year-old groups. In this study, immunohistological staining for CD138 was performed by endometrial histology, and cases in which positive cells were detected were considered to have chronic endometritis. Further, we compared the results of intrauterine microbiota examination of cases (in the follicular and luteal phases) to examine the changes in the intrauterine microbiota during the follicular and luteal phases. The means of the percentage of each bacterial taxa in the intrauterine microbiota during the follicular and luteal phases were calculated, and a 100% stacked bar graph was plotted. We statistically examined differences in the abundance of bacteria present in the uterus during the follicular and luteal phases of the study. Statistical analysis was performed using Statistical Discovery TM’s JMP^®^ (NC, USA) with t-tests and chi-square and Wilcoxon tests. *P* values <0.05 were considered statistically significant.

## Results

**Figure 1:**
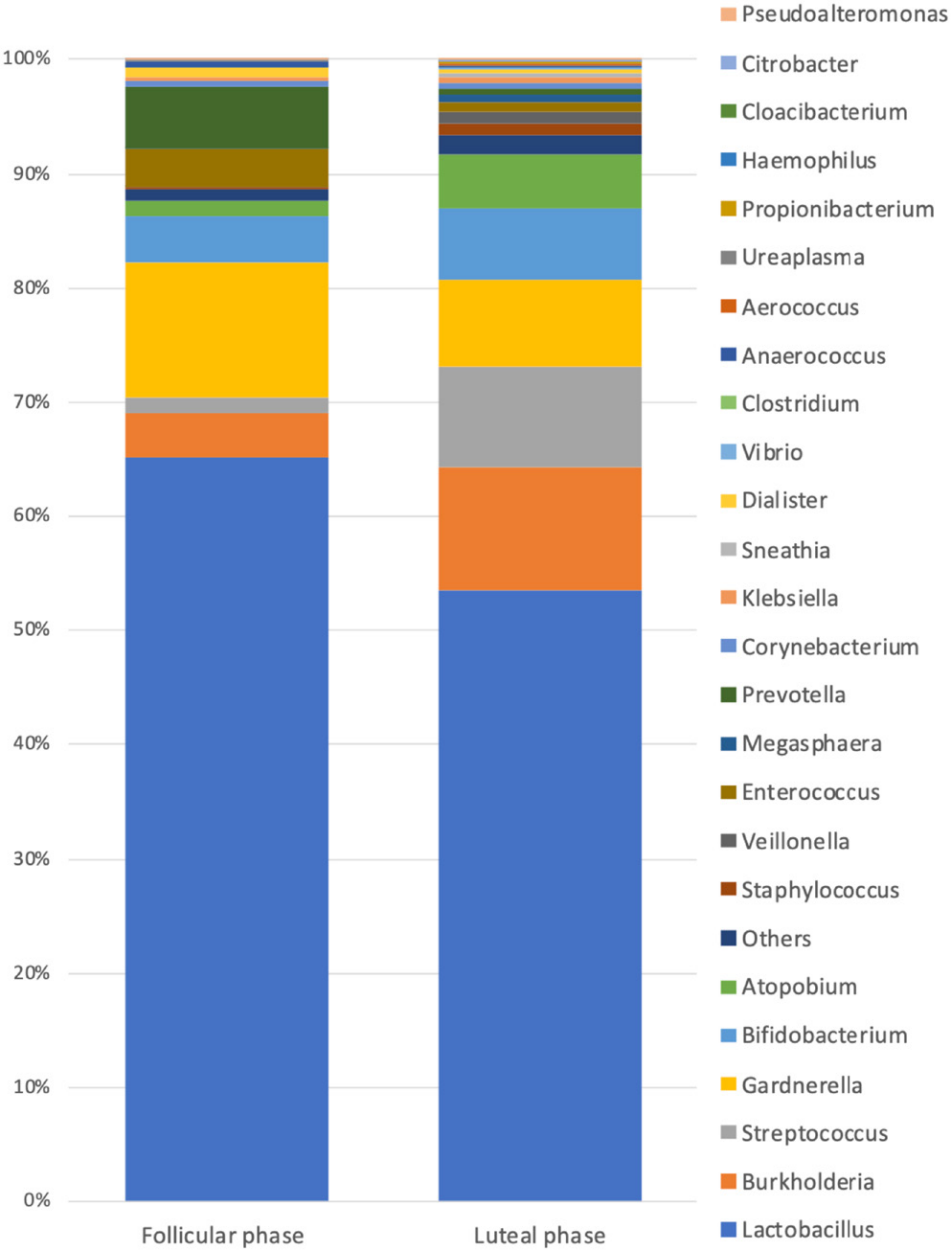
A 100% stacked bars created using the average calculated by summing the percentages of each bacterial taxa in the intrauterine microbiota of the follicular (31 patients) and luteal (116 patients) phases.

There were 116 patients whose endometriums were sampled during the luteal phase. The results comparing the patient background of the LDM and NLDM groups are shown in Table 1. No difference was observed between the two groups in terms of age, history of embryo transfer, history of pregnancy, history of miscarriage, chronic endometritis, endometriosis, endometrial polyps, serum AMH levels, and the rate of patients with high serum TSH levels. However, a trend was observed for a higher frequency of NLDM in women who had a history of childbirth (*P* = 0.0515). The results comparing the background of patients in the LDM and NLDM groups aged 26–35 years are shown in Table 2. The results of the comparison in older women aged 36–45 years are shown in Table 3. No difference was observed between the two groups in the age group below 35 years for any of the endpoints; however, in the age group of 36 years and older, a trend was observed for a higher frequency of NLDM in women who had a history of childbirth (*P* = 0.0631). Similarly, we examined the correlation between the presence or absence of chronic endometritis and the *Lactobacillus* ratio; however, no correlation was observed between the two groups (*P* = 0.2354). In the analysis of the intrauterine microbiota of cases in the follicular and luteal phases, 31 and 116 cases in the follicular and luteal phases, respectively, were studied. There were 120 different genera of bacteria identified in the womb. Among them, 19 bacteria were detected only in the luteal phase, whereas 12 were detected only in the follicular phase (Table 4). The percentages of bacterial taxa detected in each case were compared between the luteal and follicular phases by averaging them into a stacked bar graph. The results showed that the most common bacteria in the luteal phase were *Lactobacillus*, *Burkholderia*, *Streptococcus*, *Gardnerella*, *Bifidobacterium*, and *Atopobium* (in that order). Conversely, in the follicular phase, the most common bacteria were *Lactobacillus*, *Gardnerella*, *Prevotella*, *Bifidobacterium*, *Burkholderia*, and *Escherichia* (in that order) (Fig. 1). The percentage of each bacteria in the luteal and follicular phases was compared. The results showed that the percentage of *Prevotella* decreased significantly during the luteal phase (*P* = 0.0007); however, no significant change was observed in the other bacteria.

**Table 1:** Background of the two groups (LDM vs NLDM) for all ages. Data are presented as *n* (%) or mean ± S.D. Statistically significant value is in bold.

	LDM	NLDM	*P* value
No. of patients	48	68	–
Age (years)	38.5 ± 4.45	38.4 ± 3.44	0.8659^a^
Previous embryo transfer	1.52 ± 0.68	1.37 ± 0.71	0.2404^a^
Multigravida patients	21 (43.8%)	35 (51.5%)	0.3691^b^
Multipara patients	8 (16.7%)	22 (32.4%)	**0.0515**^b^
Patients with miscarriage	18 (37.5%)	18 (26.5%)	0.2252^b^
Patients with chronic endometriosis	5 (10.4%)	14 (20.6%)	0.3598^b^
Patients with endometriosis	3 (6.3%)	4 (5.9%)	0.9347^b^
Patients with endometrial polyp	5 (10.4%)	4 (5.9%)	0.3746^b^
Serum AMH (ng/mL)	3.19 ± 2.94	3.61 ± 2.65	0.4300^a^
Serum TSH >2.50 (μIU/mL)	9 (18.8%)	10 (14.7%)	0.4809^b^
% of endometrial Lactobacillus*	99.4 (92.3–100)	5.8 (0.0–89.7)	-

^a^Student’s t-test; ^b^Chi-square test; *Values are presented as median (range).

AMH, anti-mullerian hormone; LDM, *Lactobacillus*-dominated microbiota; NLDM, non--dominated microbiota; TSH, thyroid stimulating hormone.

**Table 2:** Background of the two groups (LDM vs NLDM): 26–35 years old. Data are presented as (%) or as mean ± S.D.

	LDM	NLDM	*P* value
No. of patients	11	13	–
Previous embryo transfer	1.28 ± 0.72	1.21 ± 0.72	0.3130^a^
Multigravida patients	4 (36.3%)	3 (23.0%)	0.4755^b^
Multipara patients	1 (9.0%)	2 (15.4%)	0.6423^b^
Patients with miscarriage	3 (27.2%)	1 (7.7%)	0.1997^b^
Patients with chronic endometriosis	2 (18.1%)	3 (23.1%)	0.8381^b^
Patients with endometriosis	0	0	–
Patients with endometrial polyp	2 (18.1%)	1 (7.7%)	0.4374^b^
Serum AMH, ng/mL	3.33 ± 2.93	3.33 ± 2.68	0.7677^a^
Serum TSH >2.50 μIU/mL	3 (27.2%)	3 (23.1%)	0.7085^b^
% of endometrial Lactobacillus*	99.0 (93.5–99.9)	20.7 (0.1–89.6)	–

^a^Student’st-test; ^b^Chi-square test; *Values are presented as median (range)

AMH, anti-mullerian hormone; CE, chronic endometritis; ET, embryo transfer; LDM, *Lactobacillus*-dominated microbiota; NLDM, non-*Lactobacillus*-dominated microbiota; TSH, thyroid stimulating hormone.

**Table 3:** Background of the two groups (LDM vs NLDM): 36–45 years old. Data are presented as (%) or as mean ± S.D. Statistically significant value is presented in bold.

	LDM	NLDM	*P* value
No. of patients	37	55	–
Previousembryo transfer	1.60 ± 0.58	1.48 ± 0.62	0.3129^a^
Multigravida patients	17 (%)	32 (%)	0.2108^b^
Multipara patients	7 (%)	20 (%)	** 0.0631** *^b^
Patients with miscarriage	15 (%)	17 (%)	0.3740^b^
Patients with chronic endometriosis	3 (%)	11 (%)	0.3933)^b^
Patients with endometriosis	3 (%)	4 (%)	0.8826)^b^
Patients with endometrial polyp	3 (%)	3 (%)	0.6165)^b^
Serum AMH, ng/mL	3.25±2.91	3.60±2.69	0.4240^a^
Serum TSH >2.50 μIU/mL	6 (%)	7 (%)	0.5791)^b^
% of endometrial Lactobacillus*	99.4 (92.3–100)	5.4 (0.0–89.7)	-

*Values presented as median (range); ^a^Student’s t-test. ^b^Chi-square test.

AMH, anti mullerian hormone LDM, *Lactobacillus*-dominated microbiota; NLDM, non-*Lactobacillus*-dominated microbiota; TSH, thyroid stimulating hormone.

**Table 4:** Bacterial names and number of cases detected only during the luteal and follicular phases.

Phase/number of cases	Bacteria detected
Luteal phase	
7	*Delftia*
6	*Aerococcus*
4	*Peptoniphilius, Sneathia*
3	*Stenotrophomonas,Varibaculum*
2	*Alicyclobacillus, Aquabacterium, Bradyrhizobium, Caloramator, Propionibacterium, Rhodanobacter*
1	*Agrobacterium, Cloacibacterium, Mycoplasma, Roseburia, Sulfuritalea, Shewanella, Tsukamurella*
Follicular phase	
2	*Actinobaculum, Mobiluncus, Porphyromonas*
1	*Alicyciphilus, Actinomyces, Actinomycetospora, Calothrix, Eikenella, Limnohabitans, Micrococcus, Peptostreptococcus, Spirosoma*

## Discussion

There is no evidence for intrauterine flora testing in patients in fertility practice. This study found that infertile patients who had experienced childbirth tended to have a lower percentage of uterine *Lactobacillus*. Furthermore, it was observed that this tendency was more likely to occur over the age of 36 years. Two factors may contribute to the low levels of *Lactobacillus* in the intrauterine microbiota of women who have experienced childbirth. One is that the uterus is more open after delivery, making it more susceptible to vaginal microbiota. The other is that bacteria other than *Lactobacillus* may become established in the endometrium during the postpartum amenorrhea period when estrogen levels are low. In addition, chronic endometritis is sometimes assessed by the presence of CD138-positive cells by immunohistology or hysteroscopic findings. However, there is no consensus regarding the evaluation of chronic endometritis (Song *et al.* 2019) because of inconsistency in previous reports. In this study, the presence or absence of chronic endometritis was assessed using CD138. No correlation was observed between the *Lactobacillus* ratio and the presence of chronic endometritis, indicating that a low *Lactobacillus* ratio does not cause chronic endometritis. The vagina is maintained at a pH of about 4.5 by *Lactobacillus*, which has been reported to prevent the entry and growth of pathogenic microorganisms into the uterus (Hassold & Hunt 2001); however, it is unclear how the abundance of *Lactobacillus* in the uterus affects embryo implantation. Studies comparing intrauterine pH levels and intrauterine microbiota have reported no correlation, suggesting that the inflammatory response of the endometrium due to the low *Lactobacillus* percentage may have an impact on embryo implantation (Moreno *et al.* 2016). The transplanted embryo is reportedly the most important factor in determining the occurrence of implantation (Skarin & Sylwan 1986, Hodes-Wertz *et al.* 2012). To accurately ascertain the relationship between the intrauterine microbiota and implantation rate, it is necessary to perform pre-implantation genetic testing to remove embryos with chromosome aberrations and compare the implantation rate. Thus, age, history of pregnancy, number of embryos transferred, chronic endometritis, endometriosis, endometrial polyps, thyroid abnormalities, and differences in AMH values did not affect the percentage of *Lactobacillus* in the uterus in patients who underwent their first endometrial microbiota examination (luteal phase) without using antibiotics for 4 weeks prior to the test. Patients older than 36 years tended to have a lower percentage of *Lactobacillus* in the uterus among women who have had a previous delivery, and their intrauterine environment may be the cause of their infertility. Further investigation of the intrauterine microbiota and pregnancy rates in these patients is warranted in future infertility clinics. A comparison of the intrauterine microbiota during the follicular and luteal phases in this study suggests that the intrauterine microbiota may change with the menstrual cycle. Moreno *et al.* reported that the intrauterine microbiota is stable during the acquisition of endometrial receptivity during the luteal phase and reported the benefit of assessing the intrauterine microbiota during this phase (Moreno *et al.* 2016). Chen *et al.* compared the follicular and luteal phases and noted that the endometrial microbiota might change within the menstrual cycle (Chen *et al.* 2017). However, Kyono *et al.* compared the follicular and luteal phases of the same menstrual cycle in healthy volunteers and reported no change in the bacterial microbiota (Kyono *et al.* 2018); therefore, there is a lack of consensus on the changes in the intrauterine microbiota within the menstrual cycle. The presence of bacterial genera in this study that were only detected during the follicular and luteal phases suggests that the intrauterine microbiota may change with the menstrual cycle. The major intrauterine bacteria detected in this study were *Lactobacillus*, *Burkholderia*, *Streptococcus*, *Gardnerella*, *Bifidobacterium*, *Atopobium*, *Prevotella*, and *Escherichia*. Women with high *Lactobacillus* counts are more likely to become pregnant with infertility treatment (Moreno *et al.* 2016, Kyono *et al.* 2019). Conversely, patients with higher rates of *Gardnerella* and *Streptococcus* have been reported to have lower pregnancy rates (Moreno *et al.* 2016). In the future, it is necessary to examine the mechanism underlying the influence of each uterine bacteria on the prognosis of pregnancy. This study has some limitations. The effects of the number of people who have had sexual intercourse, presence or absence of sexual contact before testing, previous use of oral contraceptives, previous artificial insemination, and history of intrauterine manipulation were not examined. Similarly, it is possible that an adequate number of cases has not been studied in the follicular and luteal phases. Furthermore, the unavailability of reports on the relationship between the success rate of fertility treatment and the rate of raising children is an issue for future studies. Interventions for women with a low percentage of *Lactobacillus* by examining the intrauterine microbiota have not yet been established. Research on the use of probiotics for the treatment of bacterial vaginosis is ongoing (Medical Xpress Web site 2017), and with reports of improvement in 75% of patients using antimicrobials and probiotics (Kawamata *et al.* 2020), antimicrobials and probiotics may be a potential treatment option. Advances in the selection of women requiring intrauterine microbiota treatment for infertility and the development of criteria for assessing the intrauterine microbiota, as well as research on how to intervene in women with abnormal intrauterine microbiota, are expected.

## Declaration of interest

The authors declare that there is no conflict of interest that could be perceived as prejudicing the impartiality of the research reported.

## Author contribution statement

K O came up with the research design and wrote the paper. A S, R A and M S guided the research design. S Y, K T, M M, M S and Y O collected the data.
